# Supplementary material for: Human Placental Schistosomiasis—A Systematic Review of the Literature
Source: Pathogens. 2024 Jun 3;13(6):470. doi: 10.3390/pathogens13060470 (PMC11206619; doi:10.3390/pathogens13060470)
Supplement: Supplementary file 1 [file pathogens-13-00470-s001.zip › pathogens-2982603-supplementary.pdf]

Systematic Review Protocol

Study Title	Human Placental Schistosomiasis – A Systematic Review of the Literature
Objective	Review of the existing literature to summarize existing reports of microscopical confirmed cases of human placental schistosomiasis.
Version	1
Date	01.04.2024
Registration	PROSPERO
Author	Jacob Gerstenberg[1,2], Johannes Mischlinger[3,4], Benjamin Schleenvoigt[5]

1 Institute for Tropical Medicine, Eberhard-Karls University Tübingen, 72074 Tübingen, Germany; jacob.gerstenberg@posteo.de
2 Centre Recherches Médicales de Lambaréné, Lambaréné, Gabon
3 Center of Tropical Medicine, Bernhard Nocht Institute for Tropical Medicine & I. Department of Medicine University Medical Center Hamburg-Eppendorf, Hamburg, Germany ; mischlinger@bnitm.de
4 German Centre for Infection Research (DZIF), partner site Hamburg-Luebeck-Borstel, Hamburg, Germany.
5 Institute of Infectious Diseases and Infection Control, Jena University Hospital/Friedrich-Schiller-University, Am Klinikum 1 07747 Jena, Germany, benjamin.schleenvoigt@med.uni-jena.de

Table of Contents

Administrative Informations 2

Introduction 2

- Rationale 2
- Specific Objectives 2

Methods 3

- Data collection 3
- Data management 3
- Risk of Bias Assessment 3

References 4

Administrative Informations

Author contributions: Conceptualization of the research idea BTS; Drafting of the study protocol JG; Reviewing of the study protocol JG, JM and BTS

Funding: This systematic review received no external funding.

Conflicts of Interest: The authors declare no conflicts of interest.

Introduction

Rationale

Schistosomiasis is one of the most important parasitic disease affecting around 220 million people worldwide [1]. It has been estimated that approximately 40 million women of reproductive age are affected [2] which can suffer from not only from female genital schistosomiasis (FGS) but in case of pregnancy egg deposition may also appear in the placenta. Since the beginning of the 20th century there have only been few case reports and clinical trials investigating placental schistosomiasis (PS). There have been methodologic difficulties to identify infested placentas with sufficient sensitivity, as standard histopathology of the placenta can only investigate a little part of the organ. Tissue dissolution techniques may increase the sensitivity and have first been described by Sutherland *et al.* in 1965. Holtfreter *et al.* (2017) have described an improved maceration technique, which allows a complete dissolution of the placental tissue while maintaining well preserved the morphological features of the observed schistosomal eggs [3]. We therefore aim to review the current literature and give an overview of the microscopically confirmed cases that have been described in the past.

Schistosomiasis has also been known to cause anemia in pregnant women and may potentially be linked to adverse birth outcomes (ABO) in their offspring. Possible sequelae are low birth weight, small for gestational age (SGA), fetal growth restriction (FGR) and stillbirth [4,5]. The exact pathophysiological link between maternal schistosomiasis and ABO have not yet been fully understood, but suggested mechanisms include inflammation at the feto-maternal interface due to placental egg deposition and the trans-placental transfer of schistosomal antigens [4]. We therefore aim to discuss the role of PS as a possible mechanistic link between maternal schistosomiasis and ABO.

Specific Objectives

1. To review the existing literature related to PS and summarize all microscopical confirmed cases.
2. To report event rates and/or estimates of prevalence of PS in pregnancy.
3. To review and discuss the potential contribution and mechanistic links of PS to adverse birth outcomes (ABO) such as low birth weight (LBW), small for gestational age (SGA), prematurity and stillbirth.

Methods

Data collection

We will search the existing literature using the electronic databases PubMed, EMBASE and MEDLINE as search tools. The search term for identification of records is: (placenta OR placental) AND (schistosomiasis OR schistosoma OR bilharzia), which will be searched in keywords of the respective electronic databases. We will include all publications up from initiation of the databases until 1st of May 2024. There will be no limitation of inclusion respective to the language of the publications. All possible study designs will be eligible,and we will include published and peer-reviewed articles as well as abstracts of conference presentations. Furthermore, we will also include literature that was not identified in electronic databases but cited in the reviewed publications. Only publications including microscopically confirmed cases of PS in humans will be selected.

Data management

Eligibility will be assessed by two reviewers independently. For this purpose, we will export the results of the literature search (citation and abstract) from above mentioned databases. Extraction will be done individually by each of the reviewer. Potential duplicates between databases will be removed manually. After removal of duplicates, all abstracts will be screened for eligibility. Potential discrepancies between the two reviewers will be resolved by a third reviewer. If eligible, full texts will be taken into consideration.

Risk of Bias Assessment

All reports identified to be eligible will undergo a risk of bias assessment based on a tool reported by Murad et al (2018) which has been developped for systematic reviews that primarily report case reports and case series [6]. This tool includes evaluation of eight items from four domains (selection, ascertainment, causality, ,and reporting). Each item is based on a leading question and scores with either 0 or 1, and scores will be summed up into an aggregate score (range 0-8 points). The respective tool including domains and leading questions for each item is summarized in Table 1. Risk of bias will be assessed by two independent reviewers. Based on the results of the evaluated eight items, each investigator will make an overall statement of the methodological quality of each assessed report. This statement can be one out of three categories (poor, acceptable, good) and does not have to be based on the sum of the aggregated score, as not each item is of equal importance for this systematic review (e.g. questions 5-7 are designed for case reports of adverse drug side effects). The overall statement rather represents a qualitative assessment by the reviewer. Potential discrepancies will be resolved by a third reviewer.

Domain	N° Item	Leading question	Points
Selection	1	Does the patient(s) represent the whole experience of the investigator? Is the selection method of patients unclear unclear to the extent that other patients with similar presentation may not have been reported?	1
Ascertainment	2	Was the exposure adequately ascertained?	1
	3	Was the outcome adequately ascertained?	1
Causality	4	Were other alternative causes that may explain the observation ruled out?	1
	5	Was there a challenge/rechallenge phenomenon?	1
	6	Was there a dose–response effect?	1
	7	Was follow-up long enough for outcomes to occur?	1
Reporting	8	Is the case(s) described with sufficient details to allow other investigators to replicate the research or to allow practitioners make inferences related to their own practice?	1

Table 1: Tool for evaluating the methodological quality of case reports and case series, adapted from Murad et al (2018) [6]

References

1. Hotez, P.J.; Alvarado, M.; Basáñez, M.G.; Bolliger, I.; Bourne, R.; Boussinesq, M.; Brooker, S.J.; Brown, A.S.; Buckle, G.; Budke, C.M.; u.c. The Global Burden of Disease Study 2010: Interpretation and Implications for the Neglected Tropical Diseases. *PLoS Negl. Trop. Dis.* **2014**, *8*, 17, doi:10.1371/journal.pntd.0002865.
2. Friedman, J.F.; Olveda, R.M.; Mirochnick, M.H.; Bustinduy, A.L.; Elliott, A.M. Praziquantel for the Treatment of Schistosomiasis during Human Pregnancy. *Bull. World Health Organ.* **2018**, *96*, 59–65, doi:10.2471/BLT.17.198879.
3. Holtfreter, M.C.; Neubauer, H.; Groten, T.; El-Adawy, H.; Pastuschek, J.; Richter, J.; Häussinger, D.; Pletz, M.W.; Schleenvoigt, B.T. Improvement of a tissue maceration technique for the determination of placental involvement in schistosomiasis. *PLoS Negl. Trop. Dis.* **2017**, *11*, doi:10.1371/journal.pntd.0005551.
4. Freer, J.B.; Bourke, C.D.; Durhuus, G.H.; Kjetland, E.F.; Prendergast, A.J. Schistosomiasis in the first 1000 days. *Lancet Infect. Dis.* **2018**, *18*, e193–e203, doi:10.1016/S1473-3099(17)30490-5.
5. Friedman, J.F.; Mital, P.; Kanzaria, H.K.; Olds, G.R.; Kurtis, J.D. Schistosomiasis and Pregnancy. *Trends Parasitol.* **2007**, *23*, 159–164, doi:10.1016/j.pt.2007.02.006.
6. Murad, M.H.; Sultan, S.; Haffar, S.; Bazerbachi, F. Methodological quality and synthesis of case series and case reports. *Evid. Based. Med.* **2018**, *23*, 60–63, doi:10.1136/bmjebm-2017-110853.

PRISMA 2020 Checklist

Section and Topic	Item #	Checklist item	Location where item is reported
**TITLE**			
Title	1	Identify the report as a systematic review.	Line 2
**ABSTRACT**			
Abstract	2	See the PRISMA 2020 for Abstracts checklist.	
**INTRODUCTION**			
Rationale	3	Describe the rationale for the review in the context of existing knowledge.	L. 58-73
Objectives	4	Provide an explicit statement of the objective(s) or question(s) the review addresses.	L. 69-73
**METHODS**			
Eligibility criteria	5	Specify the inclusion and exclusion criteria for the review and how studies were grouped for the syntheses.	L. 80-82
Information sources	6	Specify all databases, registers, websites, organisations, reference lists and other sources searched or consulted to identify studies. Specify the date when each source was last searched or consulted.	L. 75-80
Search strategy	7	Present the full search strategies for all databases, registers and websites, including any filters and limits used.	L. 76-77
Selection process	8	Specify the methods used to decide whether a study met the inclusion criteria of the review, including how many reviewers screened each record and each report retrieved, whether they worked independently, and if applicable, details of automation tools used in the process.	L. 82-83
Data collection process	9	Specify the methods used to collect data from reports, including how many reviewers collected data from each report, whether they worked independently, any processes for obtaining or confirming data from study investigators, and if applicable, details of automation tools used in the process.	L. 75-82
Data items	10a	List and define all outcomes for which data were sought. Specify whether all results that were compatible with each outcome domain in each study were sought (e.g. for all measures, time points, analyses), and if not, the methods used to decide which results to collect.	L. 80
	10b	List and define all other variables for which data were sought (e.g. participant and intervention characteristics, funding sources). Describe any assumptions made about any missing or unclear information.	NA
Study risk of bias assessment	11	Specify the methods used to assess risk of bias in the included studies, including details of the tool(s) used, how many reviewers assessed each study and whether they worked independently, and if applicable, details of automation tools used in the process.	83-89
Effect measures	12	Specify for each outcome the effect measure(s) (e.g. risk ratio, mean difference) used in the synthesis or presentation of results.	NA
Synthesis methods	13a	Describe the processes used to decide which studies were eligible for each synthesis (e.g. tabulating the study intervention characteristics and comparing against the planned groups for each synthesis (item #5)).	NA
	13b	Describe any methods required to prepare the data for presentation or synthesis, such as handling of missing summary statistics, or data conversions.	NA
	13c	Describe any methods used to tabulate or visually display results of individual studies and syntheses.	NA
	13d	Describe any methods used to synthesize results and provide a rationale for the choice(s). If meta-analysis was performed, describe the model(s), method(s) to identify the presence and extent of statistical heterogeneity, and software package(s) used.	NA
	13e	Describe any methods used to explore possible causes of heterogeneity among study results (e.g. subgroup analysis, meta-regression).	NA
	13f	Describe any sensitivity analyses conducted to assess robustness of the synthesized results.	NA
Reporting bias assessment	14	Describe any methods used to assess risk of bias due to missing results in a synthesis (arising from reporting biases).	NA
Certainty assessment	15	Describe any methods used to assess certainty (or confidence) in the body of evidence for an outcome.	NA

PRISMA 2020 Checklist

Section and Topic	Item #	Checklist item	Location where item is reported
**RESULTS**			
Study selection	16a	Describe the results of the search and selection process, from the number of records identified in the search to the number of studies included in the review, ideally using a flow diagram.	L.s 94-109
	16b	Cite studies that might appear to meet the inclusion criteria, but which were excluded, and explain why they were excluded.	L. 102-104 + 138-142
Study characteristics	17	Cite each included study and present its characteristics.	L. 119-165/Table 1
Risk of bias in studies	18	Present assessments of risk of bias for each included study.	Table 1
Results of individual studies	19	For all outcomes, present, for each study: (a) summary statistics for each group (where appropriate) and (b) an effect estimate and its precision (e.g. confidence/credible interval), ideally using structured tables or plots.	Table 1
Results of syntheses	20a	For each synthesis, briefly summarise the characteristics and risk of bias among contributing studies.	NA
	20b	Present results of all statistical syntheses conducted. If meta-analysis was done, present for each the summary estimate and its precision (e.g. confidence/credible interval) and measures of statistical heterogeneity. If comparing groups, describe the direction of the effect.	NA
	20c	Present results of all investigations of possible causes of heterogeneity among study results.	NA
	20d	Present results of all sensitivity analyses conducted to assess the robustness of the synthesized results.	NA
Reporting biases	21	Present assessments of risk of bias due to missing results (arising from reporting biases) for each synthesis assessed.	NA
Certainty of evidence	22	Present assessments of certainty (or confidence) in the body of evidence for each outcome assessed.	NA
**DISCUSSION**			
Discussion	23a	Provide a general interpretation of the results in the context of other evidence.	L. 169-176
	23b	Discuss any limitations of the evidence included in the review.	L. 175-176
	23c	Discuss any limitations of the review processes used.	-
	23d	Discuss implications of the results for practice, policy, and future research.	L. 239-247
**OTHER INFORMATION**			
Registration and protocol	24a	Provide registration information for the review, including register name and registration number, or state that the review was not registered.	NA
	24b	Indicate where the review protocol can be accessed, or state that a protocol was not prepared.	L. 91-92
	24c	Describe and explain any amendments to information provided at registration or in the protocol.	NA
Support	25	Describe sources of financial or non-financial support for the review, and the role of the funders or sponsors in the review.	L. 252
Competing interests	26	Declare any competing interests of review authors.	L. 254
Availability of data, code and other materials	27	Report which of the following are publicly available and where they can be found: template data collection forms; data extracted from included studies; data used for all analyses; analytic code; any other materials used in the review.	NA

From: Page MJ, McKenzie JE, Bossuyt PM, Boutron I, Hoffmann TC, Mulrow CD, et al. The PRISMA 2020 statement: an updated guideline for reporting systematic reviews. BMJ 2021;372:n71. doi: 10.1136/bmj.n71
